# Biofeedback in rehabilitation

**DOI:** 10.1186/1743-0003-10-60

**Published:** 2013-06-18

**Authors:** Oonagh M Giggins, Ulrik McCarthy Persson, Brian Caulfield

**Affiliations:** 1 Clarity Centre for Sensor Web Technologies, University College Dublin, Belfield, Dublin 4, Ireland; 2 School of Public Health, Physiotherapy and Population Science, University College Dublin, Belfield, Dublin 4, Ireland

**Keywords:** Biofeedback, Rehabilitation, Exercise

## Abstract

This paper reviews the literature relating to the biofeedback used in physical rehabilitation. The biofeedback methods used in rehabilitation are based on biomechanical measurements and measurements of the physiological systems of the body. The physiological systems of the body which can be measured to provide biofeedback are the neuromuscular system, the respiratory system and the cardiovascular system. Neuromuscular biofeedback methods include electromyography (EMG) biofeedback and real-time ultrasound imaging (RTUS) biofeedback. EMG biofeedback is the most widely investigated method of biofeedback and appears to be effective in the treatment of many musculoskeletal conditions and in post cardiovascular accident (CVA) rehabilitation. RTUS biofeedback has been demonstrated effective in the treatment of low back pain (LBP) and pelvic floor muscle dysfunction. Cardiovascular biofeedback methods have been shown to be effective in the treatment of a number of health conditions such as hypertension, heart failure, asthma, fibromyalgia and even psychological disorders however a systematic review in this field has yet to be conducted. Similarly, the number of large scale studies examining the use of respiratory biofeedback in rehabilitation is limited. Measurements of movement, postural control and force output can be made using a number of different devices and used to deliver biomechanical biofeedback. Inertial based sensing biofeedback is the most widely researched biomechanical biofeedback method, with a number of studies showing it to be effective in improving measures of balance in a number of populations. Other types of biomechanical biofeedback include force plate systems, electrogoniometry, pressure biofeedback and camera based systems however the evidence for these is limited. Biofeedback is generally delivered using visual displays, acoustic or haptic signals, however more recently virtual reality (VR) or exergaming technology have been used as biofeedback signals. VR and exergaming technology have been primarily investigated in post-CVA rehabilitation, however, more recent work has shown this type of biofeedback to be effective in improving exercise technique in musculoskeletal populations. While a number of studies in this area have been conducted, further large scale studies and reviews investigating different biofeedback applications in different clinical populations are required.

## Background

Biofeedback has been used for more than fifty years in rehabilitation to facilitate normal movement patterns after injury [1]. It is the technique of providing biological information to patients in real-time that would otherwise be unknown. This information can sometimes be referred to as augmented or extrinsic feedback, that is feedback that provides the user with additional information, above and beyond the information that is naturally available to them as opposed to the sensory (or intrinsic) feedback that provides self-generated information to the user from various intrinsic sensory receptors [2].

Biofeedback usually involves measurement of a target biomedical variable and relaying it to the user using one of two strategies;

1. Direct feedback regarding the measured variable, as in the case of heart rate or heart rate variability, where a numerical value is displayed on a wearable device, such as a watch.

2. Transformed feedback regarding the measured variable, where the measurements are used to control an adaptive auditory signal, visual display or tactile feedback method.

Providing patients and indeed clinicians with biofeedback during rehabilitation can have potential therapeutic effects as it may enable users to gain control of physical processes previously considered an automatic response of the autonomic nervous system [3]. In doing so it may offer the opportunity to improve accuracy during functional tasks, increase patient engagement in their rehabilitation and reduce the need for ongoing contact with healthcare professionals to monitor implementation of rehabilitation programmes.

The majority of biofeedback research has focused on the effects of biofeedback therapy in the treatment of upper limb and lower limb motor deficits in neurological disorders. Traditionally biofeedback is presented to the patient and the clinician via visual displays, acoustic or vibrotactile feedback. A recent development in rehabilitation is exercising in a gaming or virtual reality (VR) environment, thus providing a novel form of immersive biofeedback. With VR the measured patient activity is fed back via graphical or audiovisual animations providing a realistic impression to the patient [4].

The purpose of this paper is to review the biofeedback therapies that are currently being used in physical rehabilitation. This review will highlight and critique the pertinent research in this field and will identify any gaps in the existing literature. In addition, this paper will classify the different types of biofeedback that are currently being used in rehabilitation. A recent literature review by Huang and colleagues reviewed biofeedback therapies and the recent developments in this field, however no classification for the different types of biofeedback was presented [5]. To the authors’ best knowledge, this is the first paper to present such a classification system.

### Categories of biofeedback used in physical rehabilitation

**Figure 1:**
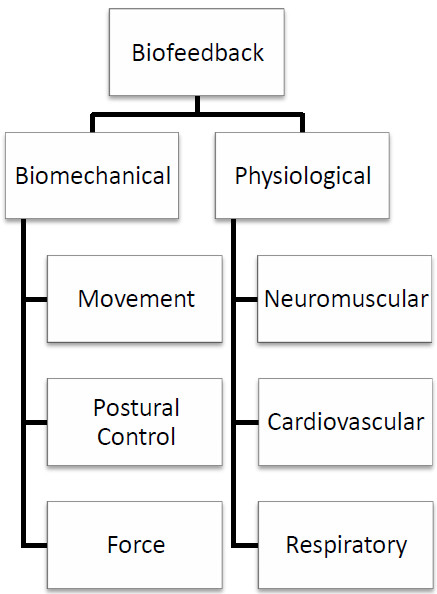
Categories of biofeedback used in physical rehabilitation.

The biofeedback measurements which are frequently used in physical rehabilitation can be categorised as being either physiological or biomechanical (Figure 1). The physiological systems of the body which can be measured to provide biofeedback are the neuromuscular system, the respiratory system and the cardiovascular system, while biomechanical biofeedback involves measurements of movement, postural control and force. Biofeedback may also be classified according to the type of signal used, however for the purpose of this review, the classification presented in Figure 1 will be used.

Other types of biofeedback which exist, such as electroencephalography, which provides information on brain wave activity and galvanic skin response, which measures skin conductance, are outside the scope of this review and therefore will not be discussed. This review will also only discuss real-time biofeedback applications, therefore offline applications, such as blood pressure monitoring have not been addressed.

## Physiological biofeedback

### Neuromuscular biofeedback

The neuromuscular system is the nervous and musculoskeletal system working together to produce movement. Any measure of these systems can be used to provide neuromuscular biofeedback. Neuromuscular biofeedback methods used in physical rehabilitation include EMG biofeedback and real time ultrasound imaging (RTUS) biofeedback.

#### Electomyography (EMG) biofeedback

EMG biofeedback is a method of retraining muscle by creating new feedback systems as a result of the conversion of myoelectrical signals in the muscle into visual and auditory signals [6]. EMG uses surface electrodes to detect a change in skeletal muscle activity, which is then fed back to the user usually by a visual or auditory signal. EMG biofeedback can be used to either increase activity in weak or paretic muscle or it can be used to facilitate a reduction in tone is a spastic one. EMG biofeedback has been shown to be useful in both musculoskeletal and neurological rehabilitation.

Draper and Ballard [7] suggested that EMG biofeedback is more effective in facilitating the recovery of quadriceps femoris muscle peak torque than electrical stimulation treatment in participants post anterior cruciate ligament reconstruction. Early reports highlighted EMG biofeedback as an efficacious therapeutic modality following menisectomy [8]. A recent randomized, single blind, clinical study suggested that the addition of EMG biofeedback to a conventional exercise programme resulted in a significantly shorter time in using a walking aid compared to conventional exercise training alone following arthroscopic partial meniscectomy [9]. In addition, the EMG biofeedback group demonstrated significantly better quadriceps femoris muscle strength and Lysholm Knee Scoring Scale scores than the home exercise and electrical stimulation groups. Similar results were found by Kirnap et al. [10], who also compared the effects of home exercise and EMG biofeedback after arthroscopic meniscectomy and found a significant increase in Lysholm Knee Scoring Scale score, knee flexion angle, quadriceps femoris muscle activity and power in the group which received EMG biofeedback. However a recent study which compared a strengthening exercise program with EMG biofeedback to the same exercise program with no biofeedback demonstrated no significant additive effect of EMG biofeedback in participants with knee osteoarthritis [11]. While the use of EMG biofeedback in these populations appears promising, further work is required as the studies discussed were small scale studies.

EMG biofeedback has been advocated by McConnell [12] as a training procedure that could be used during quadriceps exercises to equalize vastus medialis and vastus lateralis muscle activity. However the value of EMG biofeedback in the treatment of patellofemoral pain syndrome is questionable. Early reports [13] suggested that EMG biofeedback coupled with an exercise programme was an effective treatment in patients with patellofemoral pain syndrome, however Dursun and colleagues [14] found that EMG biofeedback did not produce further clinical improvement when compared with a conventional exercise program. Yip and Ng [15] also demonstrated that the addition of EMG biofeedback on vastus medialis obliquus activity had no measurable effect after treatment. More recently however, NG and colleagues [16] found that EMG biofeedback was an effective adjunct to therapeutic exercise for patients with patellofemoral pain syndrome. Further work examining the application of EMG biofeedback in the treatment of patellofemoral pain is warranted to investigate these conflicting reports.

The results of three randomised controlled trials (RCTs) suggest that EMG biofeedback may be useful in conditions of muscular tension to facilitate a reduction in neck muscle activation and therefore decrease pain. Dellve and colleagues [17] in their RCT of female workers on long-term sick leave with chronic neck pain found that an EMG biofeedback intervention was associated with increased vitality and increased performance in functional testing. More recently, Ma et al. [18], compared EMG biofeedback, active exercise, passive treatment and a no treatment control in the treatment of work-related neck and shoulder pain. The results of this study suggest that EMG biofeedback produced a generalized relaxation effect in the neck and shoulder muscles, which was not found in the other intervention groups. Voerman and colleagues [19] compared ambulant EMG biofeedback and ergonomic counselling to ergonomic counselling alone on work-related neck and shoulder pain and disability and found that while both groups showed significant reductions in self reported pain intensity and disability there was however no difference between the groups. These positive findings advocate the use of EMG biofeedback to facilitate a reduction in muscular tension and therefore decrease pain.

The efficacy of EMG biofeedback as a treatment for individuals with chronic whiplash-associated disorders is however vague. Voerman et al. [20] in a small clinical study, observed that four weeks of ambulant EMG biofeedback training for the upper trapezius muscles may be beneficial in reducing pain and disability levels and normalizing muscle activation patterns in chronic whiplash-associated disorder participants. The results of the study conducted by Ehrenborg and Archenholtz [21] however did not support the use of EMG biofeedback of the upper trapezius muscle as an adjunct to an interdisciplinary rehabilitation programme for people with chronic whiplash-associated disorders.

Extensive research has been conducted examining the efficacy of EMG biofeedback in the rehabilitation of patients with hemiplegia following cardiovascular accident (CVA) [22-29]. Armagan and colleagues [22] demonstrated the potential benefits of EMG biofeedback in conjunction with exercise in maximising hand function in hemiplegic patients. The results of a study performed by Aiello and colleagues [23] suggested that treadmill gait retraining augmented with EMG biofeedback facilitates improvements in gait function in post CVA participants. Inglis et al. [24] showed that compared to conventional therapy, EMG biofeedback resulted in greater improvements in functional properties such as muscle force, active range of movement and motor recovery in hemiplegic patients. However a systematic review concluded that EMG biofeedback has no effect in improving joint range of motion, functional ability, or stride length or gait speed following CVA [30], however there is some evidence of improvements in gait quality assessments, range of motion at the shoulder and restoration of motor power. Further work [25-27] has been conducted in this field since the publication of this review, therefore further systematic reviewing of this topic is warranted.

Several researchers have investigated the application of EMG biofeedback to modify gait in children with cerebral palsy (CP). Early reports suggest that EMG biofeedback of triceps surae muscle activity during gait may be efficacious in improving gait symmetry in children with CP [31]. A preliminary study showed that two children diagnosed with CP who used EMG biofeedback demonstrated improved toe clearance during the swing phase of gait and a newly learned ability to recruit and relax the anterior tibialis [32]. Dursun and colleagues [33] also evaluated the effectiveness of biofeedback treatment on gait function in children with CP. This larger study included thirty-six children with cerebral palsy who were randomly assigned to receive either EMG biofeedback and exercise or exercise only. The results of this study showed that children who received biofeedback showed significant improvements in muscle tone and ankle range of movement, compared to children who received the exercise programme only. Gait showed statistically significant progress in both groups, but the biofeedback group was superior to the exercise only group. A recent study by Bloom and colleagues [34] examined whether the prolonged use of EMG biofeedback could improve upper extremity function in children with CP. This small investigation demonstrated promising results, suggesting that further testing of prolonged EMG biofeedback in this population is warranted.

EMG biofeedback is the most widely used and widely reported method of biofeedback. However, the limited number of large RCTs and systematic reviews means further work is required. Nevertheless the existing evidence for the use of EMG biofeedback in musculoskeletal and neurological rehabilitation appears promising.

### *Real*-*time ultrasound Imaging* (*RTUS*) *biofeedback*

RTUS send short pulses of ultrasound into the body and using reflections received from tissue interfaces, images of internal structures are produced [35] thus RTUS is capable of giving immediate real-time visual feedback of muscle activity by allowing the user to directly see the muscle changing shape/length on a display. A recent survey concluded that 81% of physiotherapist using RTUS used it as a biofeedback tool during rehabilitation [36]. It has been found that augmenting typical clinical instruction with visual feedback of the anterolateral abdominal wall using RTUS reduced the number of trials needed for subjects with [37] and without [38] low back pain (LBP) to perform the abdominal hollowing exercise. Conversely Teyhen and colleagues [39] reported that the addition of RTUS biofeedback did not enhance the ability of participants with LBP to perform the abdominal hollowing exercise over those who had not received biofeedback. Reports suggest that RTUS used to provide visual biofeedback improves activation of the multifidus muscle in healthy subjects [40]. RTUS has also been successfully used to provide visual feedback of pelvic floor muscle activation. Dietz et al. [41] showed that 32 of 56 women learned correct activation of their pelvic floor muscles with less than 5 minutes of RTUS biofeedback training. Ariail et al. [42] reported on the use of RTUS in the retraining of the pelvic floor muscle in a single case postpartum and concluded that the use of RTUS was a helpful biofeedback tool for re-education and rehabilitation of the pelvic floor muscles for this patient. While RTUS is widely used in physical medicine and rehabilitation, further large RCTs are required to examine its role as a biofeedback tool in physical rehabilitation.

## Cardiovascular biofeedback

Cardiovascular measures which can be used to provide real time biofeedback include heart rate (HR) and heart rate variability (HRV). Blood pressure and skin temperature are offline methods of biofeedback and therefore will not be discussed here.

### Heart rate (HR) biofeedback

HR can be measured using a heart rate monitor or an electrocardiogram to deliver feedback to patients. HR biofeedback is a therapeutic approach which allows patients to control their HR by means of direct representation of the numerical value of HR on a wearable device such as a watch or a handheld display. Early studies suggest that HR biofeedback could significantly lower mean HR and systolic blood pressure during treadmill exercise [43]. The results of a small study by Fredrikson and Engel [44] found that HR biofeedback resulted in a significant decrease in HR while exercising on a cycle ergometer, however systolic blood pressure was unaffected by the feedback. More recently, Moleiro and Cid [45] investigated the effects of HR biofeedback training on the control of HR during a physical exercise test, comparing it to verbal instructions to reduce HR. They found that the participants who trained with HR biofeedback showed a greater attenuation in the increase in HR produced by exercise than participants who trained with verbal control instructions. Recently HR biofeedback has been investigated as a means of controlling blood pressure in untreated hypertensives. This pilot investigation examined the effect of a short HR biofeedback protocol on the control of blood pressure and found that the systolic blood pressure and mean arterial pressure responses to an emotional speech test were significantly smaller in the biofeedback training group than in the control group who underwent blood pressure monitoring [46]. HR is a widely used measure in clinical practice, however the research that exists to support the use of HR biofeedback is limited.

### Heart rate variability (HRV) or respiratory sinus arrythmia (RSA) biofeedback

HRV refers to the variability in the time between heart beat. These variations in HR are regulated by the autonomic nervous system. HRV at the frequency of respiratory, which is also termed RSA, refers to the increase in HR with inspiration and the decrease in HR with expiration [47]. HRV is easily measured and relatively reliable and thus it has been used as an index to understand a person’s internal state [48]. HRV and RSA both provide biofeedback on the cardiovascular system and both terms are used interchangeably in the literature. HRV biofeedback appears to be a useful adjunct in the treatment of asthma and may help to reduce dependence on steroid medications [49]. Preliminary data suggests that HRV biofeedback can be used to improve overall functioning and depression in patients with fibromyalgia [50]. Giardino et al. [47] examined the efficacy of an HRV biofeedback and pulse oximetry biofeedback intervention on functioning and quality of life in patients with chronic obstructive pulmonary disease. Promising findings from this report include improvements in walking and quality of life markers, however this intervention was not compared to a control therefore further investigations are necessary to draw conclusions. Research with clinically depressed individuals indicates that RSA biofeedback training facilitates an increase in HRV amplitude and a decrease in depressive symptoms [51]. Preliminary evidence also exist to support the efficacy of RSA biofeedback in improving physiological and psychological health for individuals with posttraumatic stress disorder [52]. Reports also support the efficacy of HRV biofeedback in improving symptoms and quality of life in patients with coronary heart disease [53]. A similar study by Luskin et al. [54], demonstrated that eight sessions of HRV biofeedback produced reductions in perceived stress and improved function on the 6-minute walk test in patients with heart failure. While HRV/RSA biofeedback is a relatively new area of research, preliminary observations suggest that it may be useful in improving symptoms and quality of life in a range of health conditions.

## Respiratory biofeedback

Respiratory biofeedback is delivered by measuring breathing using electrodes or sensors attached to the abdomen and by converting breathing to auditory and visual signals for the user. Teaching diaphragmatic breathing in patients with respiratory disease is the most common means of providing respiratory biofeedback. Reports suggest that biofeedback assisted diaphragmatic breathing and systematic relaxation were equally as effective as propranolol in reducing the frequency, severity and duration of migraine headaches after six months of treatment [55]. Delk et al. [56] compared diaphragmatic excursion and EMG feedback of accessory muscle activity to a control intervention of temperature biofeedback combined with relaxation therapy in participants with cystic fibrosis. Results of this study revealed significant improvements in measures of lung function in the experimental group while the control group showed no change.

Biofeedback on breathing exercises has been shown to be an effective treatment for hypertension. Grossman and colleagues [57] investigated the effects that breathing exercises guided by interactive music feedback had on hypertension in their RCT and found this intervention to be effective in reducing blood pressure. The results of a double blind RCT conducted by Schein et al. [58] also report the efficacy of breathing control exercises guided by interactive music feedback in reducing blood pressure in hypertensive participants. The same respiratory biofeedback tool has also been shown to be helpful in reducing the anxiety experienced by individuals visiting the dentist. Morarend and co-workers [59] established from their RCT that the use of breathing exercises guided by interactive music feedback resulted in a significant reduction in the negative feelings associated with dental injections.

Respiratory biofeedback has been suggested as a useful tool for calming down breathing and for promoting relaxation [60]. Kapitza et al. [60] compared the effect of breathing exercises guided by placebo respiratory biofeedback to real respiratory biofeedback in a group of participants with chronic LBP. The intervention group received ordinary, synchronized feedback of their own breathing excursions, whereas the control group received no feedback, but a constant proxy signal corresponding to a breathing rate of approximately eight breaths per minute. While higher reductions in pain levels were noted at rest and during activity in the treatment group there was no significant differences between the groups at follow up. Results from a RCT also indicate that respiratory biofeedback training aimed at regularising breathing pattern is an effective therapeutic in the treatment of panic disorders [61].

Respiratory biofeedback is used to slow down the respiratory rate and hence promote relaxation. Reports suggest that biofeedback of breathing may be efficacious in the treatment of a number of conditions, however more extensive research is required in this field.

## Biomechanical biofeedback

Biomechanical biofeedback involves measurements of the movement, postural control and forces produced by the body. Inertial sensors, force plates, electrogoimeters, pressure biofeedback units and camera based systems are all measurement devices which can be used to provide biomechanical biofeedback. Biomechanical biofeedback is more complex than physiological biofeedback as, one measurement device can be used to deliver different types of biomechanical feedback. For example, a force plate can be used to deliver both feedback on force and postural control. While the previous section on physiological biofeedback was presented according to the physiological systems measured, this section on biomechanical biofeedback will be discussed according the measurement instruments used.

### Inertial sensors

Inertial sensing uses accelerometers and gyroscopes to estimate three-dimensional (3-D) kinematic information of a body segment, such as orientation, velocity and gravitational force. An accelerometer measures acceleration and gravitational acceleration, while a gyroscope is used to measure angular velocity [62]. These inertial sensor parameters are used as input to a feedback system that delivers a wide variety of forms of feedback to the user including, auditory, visual and tactile signals. As a result of their small size and portability inertial sensors have proven useful in movement and balance applications.

A number of researchers have investigated the role of inertial based sensing biofeedback in balance training. Davis and colleagues [63] used gyroscopic measurements to provide biofeedback and found significant changes in trunk angular displacement in both young and older participants during a number of balance tasks compared to control treatment. Verhoeff et al. [64] also examined the effects of a gyroscopic biofeedback system on trunk sway during dual tasking (performing a cognitive and a motor task) while walking. Similar to Davis and Colleagues they enrolled both elderly and young participants in their study and found that the young participants were able to react to the biofeedback while walking and performing a dual task at the same time. The elderly reduced their trunk sway with biofeedback while walking normally however, when a cognitive or a motor task was added, they were less able to react to the biofeedback and reduce trunk sway. Inertial based sensing biofeedback has also been evaluated in clinical populations. Dozza and colleagues [65,66] evaluated the effectiveness of using an audio biofeedback system based on accelerometric sensors for improving postural stability and balance in healthy subjects and in patients with bilateral vestibular loss. The first study showed that using the audio biofeedback system significantly influenced participants’ balance. The results of the study including participants with bilateral vestibular loss indicated that the audio biofeedback training reduced postural sway and was more effective for participants with bilateral vestibular loss than for the unaffected controls. A recent study by Nicolai and colleagues [67] studied whether an audio biofeedback system could be used to enhance postural control in participants with the neurological condition, progressive supranucular palsy. While improvements were demonstrated in balance, there were no significant improvements noted in measures of function. The same research group [68] more recently, investigated whether the same biofeedback system could be used to enhance postural control in participants with Parkinson’s disease. While they did observe improvements in both measures of balance and function, similar to the previous report, the results were from a small uncontrolled study with only seven participants. Soon et al. [69] examined the efficacy of using an inertial-based sensing modality as biofeedback during a tandem stance task in stroke patients. Compared with the control group, participants who received knowledge of performance biofeedback with the inertial-based sensors improved both average trunk angular displacement and velocity in trunk sway during tandem stance. While this was a small study, including only six participants per group, the improvements were shown to persist one month after the intervention.

Research has shown that sensor based feedback can be used to modify movement or behaviour. Breen et al. [70] used a biofeedback system which used a single accelerometer to correct neck posture in computer users. Crowell and colleagues [71] found that individuals can use real-time feedback of tibial acceleration from an accelerometer to reduce loading on their lower extremities while running and they can maintain the reductions for at least ten minutes after the feedback is removed.

Inertial measurement units have also been used to monitor physical activity. Much work has been conducted evaluating pedometers as a means of improving exercise compliance in people with diabetes, obesity and congestive heart failure [72-74]. Accelerometers are particularly useful in providing objective feedback of ambulatory activity to investigators and study participants in exercise adherence research [75]. Koizumi et al. [76] evaluated the efficacy of accelerometer-based feedback on daily physical activity in community-dwelling older women and found that using accelerometers can significantly improve the quantity and quality of daily physical activity as well as cardiorespiratory endurance.

Inertial sensors have gained popularity due to their small size and portability, making them suitable for use outside the laboratory setting. While further research is required, preliminary reports have used inertial sensor biofeedback effectively to retrain balance, to modify movement and to monitor physical activity.

#### Force plate systems

Force plate systems measure the ground reaction force generated by the body and can be used to give feedback on balance, movement and gait. The feedback is normally delivered by using the ground reaction forces as input to a visual display that changes with changes in force. A number of investigators have used force plate biofeedback to improve symmetry in standing posture, weight bearing status or balance and to train an awareness of movement. The efficacy of using a force platform biofeedback system to improve balance in CVA rehabilitation has been examined. Reports suggest that visual feedback training using a force plate system is an effective method to gain symmetrical stance following CVA [77,78] with no associated improvements in overall postural sway [79,80]. Barclay- Goddard et al., concluded after systematic reviewing of seven randomized controlled trials that force platform biofeedback did not improve clinical measures of balance however significant improvements in laboratory force platform indicators of stance symmetry were found for regimens using auditory and visual feedback [81]. A more recent systematic review by Van Peppen and colleagues [82] concluded that the addition of visual feedback therapy in bilateral standing following CVA shows no statistically significant effects on symmetry of weight distribution between paretic and non-paretic leg, postural sway in bilateral standing, gait and gait-related activities compared with conventional therapy [82].

Others have investigated the efficacy of force plate biofeedback training in improving gait symmetry in different populations. White and Lifeso [83] evaluated the effects that ground reaction force (GRF) feedback had in reducing asymmetric limb loading after total hip arthroplasty and concluded that real-time visual feedback is an effective method of teaching total hip arthroplasty patients to equalize limb loading. Dingwell and colleagues [84] also evaluated the effects of providing feedback of gait symmetry using a specially designed treadmill with two force plates mounted under the treadmill belt to measure GRF in trans-tibial amputees. While significant decreases in the degree of asymmetry were demonstrated after visual feedback was given, these results should be interpreted with caution due to the small sample size.

A number of investigations have evaluated the effectiveness of force platform biofeedback for training balance and mobility tasks in older populations. Shivonen et al. [85,86] showed that visual feedback based balance training using a force plate reduced the incidence of falls among frail older women living in residential care. The investigation by Hatzitaki et al. [87] demonstrated that weight shift training using a force plate system with visual feedback resulted in improvements in standing balance in community dwelling older women.

Force plate biofeedback systems have been used to retrain balance and weight bearing status post CVA and orthopaedic surgery and in older adults at risk of falls. While force plates provide accurate kinetic measurements, they are however restricted to a laboratory or clinical environment. Further research in this field is warranted due to the conflicting findings reported coupled with the small sample sizes studied.

### Electrogoniometery

Electrogoniometry allows measurement of joint kinematics during functional tasks and movements yielding real-time feedback to clinicians and patients. As the kinematics of the joint change feedback is delivered, usually via an auditory signal or visual display. Ceceli et al. [88] and Morris et al. [89] analyzed the effectiveness of providing kinematic biofeedback of the knee, using electrogoniometers compared with conventional physiotherapy in efforts to minimize genu recurvatum in participants who had a CVA. Ceceli et al. [88] found that participants who were provided with the kinematic biofeedback showed a statistically significant decrease in the number of knee hyperextensions compared with those who had received conventional physiotherapy only. In the study by Morris et al. [89] participants received treatment in two separate phases; participants in the experimental group received kinematic biofeedback during the first phase and conventional physiotherapy during the second phase. Control group participants received conventional physiotherapy during both phases. This investigation showed a moderate effect for increased gait speed in the experimental group but no effect in the control group after the first phase of treatment. While both groups demonstrated statistically significant reductions in peak knee extension during stance, there was no difference noted between the groups. Colborne et al. [90] also used an electrogoniometer to provide CVA participants with kinematic biofeedback of ankle joint movement in an attempt to improve ankle control during gait. The authors found that kinematic biofeedback training resulted in a moderate increase in gait speed while conventional physiotherapy resulted in only a small improvement. However Kuiken et al. [91] examined the effects of a computerised biofeedback knee goniometer on patients’ compliance with active range of motion exercises after total knee arthroplasty and concluded that the feedback provided by the device did not have a significant influence on the rate of exercise performance. While electrogoniometery is a relatively inexpensive method of providing kinematic biofeedback, the overall benefit of using this technology has yet to be proven.

### Pressure biofeedback unit

A pressure biofeedback unit (PBU) is a tool developed to aid the retraining of muscle activity and can provide useful visual biofeedback during treatment [92]. A PBU consists of an inflatable cushion which is connected to a pressure gage, which displays feedback on muscle activity. These units are relatively inexpensive and this technique is more easily applied in the clinical setting in comparison to previously mentioned techniques. PBUs have been used to indicate correct contraction of the transversus abdominis muscle during the abdominal hollowing exercise. Ciarns and colleagues [92] used a PBU in their study to quantify abdominal muscular dysfunction in participants with LBP or a history of LBP. PBUs have also been used to assess the deep cervical flexor muscles in individuals with and without neck pain [93]. Hudswell and colleagues [94] used a PBU in their study to measure deep neck flexor strength during the cranio-cervical flexion test. However no research comparing a PBU intervention to a control in the treatment of neck pain has been found. Research has found that lumbar spine stabilisation using a PBU results in significant increases in gluteus medius and internal oblique activity during a hip abduction exercise [95]. While the PBU is a useful tool for assessing the abdominal drawing in exercise, other research investigating this biofeedback tool is limited.

### Camera based systems

Video cameras allow clinicians and patients to examine locomotion qualitatively, whereas optical motion capture systems allow for quantitative 3-D movement analysis. Optical motion capture systems use a network of cameras to detect a series of markers placed on anatomical landmarks on a subject’s body. This information is then used by the system to deliver visual feedback of movement and posture.

Video camera feedback has been investigated in rehabilitation. Kim and colleagues [96] investigated the effects of using a video camera to provide visual feedback to participants with winged scapula during a push up exercise. Providing visual biofeedback resulted in increased activity of the serratus anterior muscle and decreased activity of the upper trapezius muscle. Research has also shown that using videotape biofeedback is an effective instructional method for enhancing motor skill acquisition in a post stroke population [97]. Despite the high accuracy to assess position, optical motion capture systems are generally restricted to a laboratory environment and a dearth of evidence exists to establish their role as a biofeedback tool in rehabilitation.

### Recent developments in biofeedback signal delivery

Biofeedback is most commonly delivered using visual, auditory or haptic signals however recent years have witnessed the emergence of immersive, VR biofeedback signals. VR and therapeutic exergames provide patients opportunities to engage in meaningful, intensive, enjoyable tasks related to real-life interests and activities of daily living [98]. A small case study reported on the use of computerized biofeedback training in a VR environment to improve hand function in a post CVA population [99]. Participants received haptic, visual and auditory feedback as they performed hand exercises, with graphics displayed on a personal computer screen. Each of the three participants studied showed improvement in measures of hand function following the two week training program. Broeren et al. [100] made use of a game in a virtual environment, along with a force-feedback haptic device, to improve control of a CVA patient’s left hemiparetic arm. Their results showed that the patient was motivated to practice and exhibited improved dexterity, grip force, and motor control. Betker et al. [101] found that centre of pressure (CoP) biofeedback controlled by video game–based exercises could improve dynamic balance control in three cases with various neurological disorders. This study also reported that the CoP-controlled video game–based exercise regime motivated subjects to increase their practice volume and attention span during training. Although these result are taken from studies with small sample sizes, the evidence suggest a role for VR based biofeedback in neurological rehabilitation. A larger study, conducted by Piron and colleagues [102] demonstrated that VR based biofeedback could enhance upper limb function in CVA participants. Subsequent to this study, Piron et al. [103] conducted a trial in which CVA participants received reinforced feedback in a virtual environment for the upper limb or a control intervention of conventional rehabilitation. Following the intervention period only the intervention group demonstrated significant improvements in measures of function. There was however no difference observed between the groups. Nevertheless this data suggests that the recovery of arm motor function in patients after recent CVA is promoted by an augmented feedback strategy, which is applied through a virtual-environment. Crosbie et al. [104] conducted a RCT to compare VR mediated upper limb therapy to standard therapy in a post CVA population. Both groups reported changes to their upper limb activity levels, however there was no difference between the groups following the intervention.

Preliminary evidence suggests that biofeedback via an exergame may be used to enhance exercise technique. Doyle et al. [105] showed that interacting with a game incorporating simple visual feedback results in improved exercise accuracy compared to performing the exercise from memory or with limited feedback in the form of an instructional video demonstration. Fitzgerald et al. [106] examined the effects of providing feedback during wobble board exercises on postural stability. Healthy adults were randomly assigned to either an exergaming group, who received visual biofeedback using a therapeutic exergaming system or to a control group, who received no feedback during exercise. While both groups in this study showed similar improvement in measures of postural stability, a greater level of interest and enjoyment was observed in the exergaming group, suggesting that there may be potential benefits to using exergaming biofeedback in the rehabilitation setting.

Recent years have witnessed huge development in the field of VR and therapeutic exergaming. Consequently VR and therapeutic exergaming biofeedback is an emerging field. The early evidence gathered was adapted from small case studies however a number of larger studies and RCTs have been conducted more recently. The existing evidence suggests a role for VR based biofeedback and exergaming biofeedback in rehabilitation, however further work is required before definite conclusions can be drawn.

## Conclusion

Biofeedback has been used for many years to assist patients and clinicians during rehabilitation. This paper has reviewed the biofeedback applications that are currently being used in physical rehabilitation and classified the different types of biofeedback into two main categories; physiological biofeedback biomechanical biofeedback. The research in this field primarily focuses on the use of biofeedback in rehabilitation of patients with neurological disorders. EMG biofeedback is by far the most popular form of biofeedback, however newer technologies are been investigated for their potential as biofeedback tools. While the evidence to support the use of biofeedback in rehabilitation appears promising, there is however a lack of systematic reviews including a large number of RCTs examining this subject. Further large RCTs and systematic reviews investigating different biofeedback applications in different clinical populations are warranted.

## Abbreviations

EMG: Electromyography; RTUS: Real time ultrasound imaging; CVA: Cardiovascular accident; LBP: Low back pain; VR: Virtual reality; RCT: Randomised controlled trial; CP: Cerebral palsy; HR: Heart rate; HRV: Heart rate variability; RSA: Respiratory Sinus Arrhythmia; 3-D: Three dimensional; GRF: Ground reaction force; PBU: Pressure biofeedback unit; CoP: Centre of pressure.

## Competing interests

The authors declare that they have no competing interests.

## Authors' contributions

OMG designed the review and drafted the manuscript under the guidance of BC. BC and UMP contributed concepts and edited and revised the manuscript. All authors read and approved the manuscript.
